# Advantages of Remimazolam in Pediatric Anesthesia: A Narrative Review

**DOI:** 10.3390/children13030348

**Published:** 2026-02-27

**Authors:** Alessandro Vittori, Cecilia Di Fabio, Elisa Francia, Ilaria Mascilini, Riccardo Tarquini, Corrado Cecchetti, Giuliano Marchetti, Franco Marinangeli, Teresa Grimaldi Capitello, Marco Cascella

**Affiliations:** 1 Department of Anesthesia, Critical Care and Pain Medicine, ARCO, Ospedale Pediatrico Bambino Gesù IRCCS, Piazza S. Onofrio 4, 00165 Rome, Italy; 2 Department of Life, Health and Environmental Sciences (MeSVA), University of L’Aquila, Piazzale Salvatore Tommasi 1, Blocco 11, 67010 L’Aquila, Italy; 3 Surgery Unit, Bios Medical Center, Via Domenico Chelini 39, 00197 Rome, Italy; 4 Clinical Psychology Unit, Department of Neuroscience, Ospedale Pediatrico Bambino Gesù IRCCS, Piazza S. Onofrio 4, 00165 Rome, Italy; 5 Department of Medicine, Surgery and Dentistry, University of Salerno, Via Salvador Allende, 43, 84081 Baronissi, Italy

**Keywords:** remimazolam, anesthesia, intensive care unit, pediatric anesthesia, sedation, non-operative room anesthesia, pain, children, opioid, anxiety

## Abstract

Remimazolam is an ultra-short-acting benzodiazepine developed according to the “soft drug” concept and characterized by rapid onset, predictable offset, organ-independent metabolism, and the availability of a specific antagonist. Due to these pharmacological features, this drug represents a particularly attractive option for pediatric anesthesia and sedation, a field in which traditional agents are often limited by hemodynamic instability, prolonged recovery, and adverse respiratory effects. This narrative review summarizes and discusses the current evidence regarding the use of remimazolam in pediatric patients, focusing on pharmacokinetics, pharmacodynamics, clinical applications, and safety. Available data indicate that remimazolam provides effective sedation and anesthesia in children across multiple settings, including induction of general anesthesia, non-operating room anesthesia, and intensive care unit sedation. Compared with propofol and midazolam, remimazolam is generally associated with greater hemodynamic stability, rapid recovery, reduced emergence delirium, and a favorable respiratory profile, while maintaining comparable efficacy. Intranasal administration has also shown promise as a premedication strategy for reducing preoperative anxiety, although it may occasionally be associated with pain. Even if remimazolam lacks intrinsic analgesic properties, its use appears to indirectly improve postoperative comfort by attenuating stress responses and emergence agitation. Despite encouraging results, pediatric use of remimazolam remains off-label in many countries, and evidence is still limited by small sample sizes and heterogeneous protocols. Further large-scale randomized controlled trials are needed to define optimal dosing strategies, long-term safety, and their definitive role in pediatric anesthetic and sedative practice.

## 1. Introduction

Remimazolam is a next-generation benzodiazepine characterized by an ultra-short pharmacokinetic (PK) profile, rapid onset of action, absence of drug accumulation, and metabolism independent of hepatic and renal function [1,2,3]. The molecule was developed by structurally combining pharmacological features of midazolam and remifentanil through the incorporation of a carboxylic ester linkage, allowing rapid hydrolysis by tissue esterases [4]. Owing to these properties, remimazolam has attracted increasing interest for use in a variety of clinical settings, including general anesthesia, procedural sedation, and, more recently, the maintenance of sedation in intensive care units [5,6].

The pediatric population represents a particularly relevant target for novel sedative agents, as many traditional drugs are associated with well-recognized limitations, such as cardiovascular depression, delayed onset, and prolonged recovery times [7,8]. Moreover, children exhibit increased vulnerability to sedative-related adverse effects, further complicating perioperative and procedural management [9,10]. In this context, remimazolam has been progressively introduced into clinical practice due to its favorable pharmacological profile, including PK features and pharmacodynamic (PD) properties such as reduced risk of respiratory depression and hemodynamic instability, and the possibility of prompt antagonism with flumazenil [11].

In recent years, several randomized and observational studies have explored the role of remimazolam in common pediatric procedures. A recent dose-exploration study demonstrated that intranasal remimazolam can rapidly and effectively reduce preoperative anxiety in children [12]. In a 2025 study involving 184 pediatric patients, Yiwen et al. reported that remimazolam significantly reduced the incidence of postoperative delirium and hemodynamic instability [13]. Similarly, Wan et al., in a comparative study including 193 pediatric patients undergoing adenotonsillectomy, showed that remimazolam was associated with faster recovery, reduced emergence agitation, greater hemodynamic and cardiocirculatory stability, and fewer adverse events [14]. Despite these encouraging findings, several studies emphasize that the use of remimazolam in pediatric patients remains off-label in many countries, highlighting regulatory and clinical uncertainties that still need to be addressed [15].

On this basis, the present narrative review aims to critically synthesize the available clinical evidence on remimazolam in pediatric patients, with a focus on its safety, efficacy, and applicability across different clinical settings. Given the marked heterogeneity in study designs, dosing regimens, and procedural contexts, a narrative approach was adopted to integrate pharmacological principles with emerging clinical data and to highlight current knowledge gaps relevant to future research and clinical practice. Studies indexed in PubMed and in English were searched.

## 2. Pharmacokinetics and Pharmacodynamics Properties

Remimazolam is a structural derivative of midazolam and is available in two salt forms, benzenesulfonate and toluenesulfonate. The introduction of a methyl propionate side chain confers susceptibility to rapid hydrolysis by carboxylesterases, primarily in the liver, resulting in elimination according to first-order kinetics and the formation of an inactive metabolite, CNS7054 [16]. From a chemical–pharmacological perspective, remimazolam was developed following the “soft drug” concept (a therapeutically active compound that is intentionally designed to be rapidly and predictably inactivated by metabolism once it has produced its desired effect) [17], incorporating an ester linkage into the benzodiazepine scaffold to ensure rapid and predictable metabolism [18]. Owing to this structural modification, remimazolam undergoes rapid hydrolysis into the inactive metabolite CNS7054 [19,20].

These metabolic characteristics clearly differentiate remimazolam from traditional benzodiazepines such as midazolam, as its metabolism depends on carboxylesterase 1 (CES1) and is largely independent of hepatic and renal function [21]. Phase I studies conducted in healthy volunteers demonstrated that, following intravenous administration, remimazolam exhibits significantly higher systemic clearance compared with midazolam (70.3 vs. 20.3 L h^−1^) and a markedly lower volume of distribution (34.8 L vs. 81.8 L). This PK profile translates into rapid elimination, with an elimination half-life of approximately 0.75 h for remimazolam compared with 4.29 h for midazolam, and a reduced propensity for tissue accumulation [22].

Remimazolam is metabolized by plasma esterases—particularly hepatic carboxylesterases—into an inactive metabolite and is predominantly excreted via the urine [23]. After intravenous administration, approximately 92% of the drug is bound to plasma proteins, mainly albumin [11]. Overall, remimazolam displays high clearance, rapid metabolism, and predictable onset and offset kinetics [2,20], allowing rapid recovery even after prolonged or continuous infusions and reducing the risk of residual sedation. These properties distinguish remimazolam from other sedatives such as midazolam and propofol, particularly in terms of minimal accumulation and limited prolonged effects [1]. As with other benzodiazepines, its sedative effects can be rapidly antagonized by flumazenil [1].

A comprehensive review published in 2025 evaluated the influence of age, renal function, and hepatic impairment on remimazolam pharmacokinetics [24]. The analysis showed no clinically relevant differences between younger and elderly individuals, between patients with normal renal function and those with end-stage renal disease (eGFR > 90 vs. <15), or between subjects with normal liver function and those with mild-to-moderate hepatic impairment. In pediatric patients, PK behavior appears broadly comparable to that observed in adults, although some data suggest an even faster pharmacodynamic response.

In a study by Gao et al. [21] investigating remimazolam pharmacokinetics following intravenous infusion in anesthetized children, the standardized central volume of distribution was reported as 7.6 L, compared with values ranging from 3.6 to 4.7 L in adult populations [16,22]. The standardized steady-state volume of distribution was 41 L in children, compared with approximately 35 L in adults [22]. Clearance in pediatric patients was 0.73 L min^−1^, slightly lower than the ~1 L min^−1^ reported in adult studies [16,22]. The causes of this difference are multiple, and are generally linked to the child’s own characteristics, to the immature nature of his excretory elements (such as the maturation of carboxylesterases).

Evidence from studies on intranasal administration further supports the rapid onset of action in children. A clinical investigation evaluating intranasal remimazolam as premedication showed that most pediatric patients achieved mild sedation within 10 min of administration [15]. The same study reported intranasal bioavailability of approximately 25–50%, consistent with adult data but potentially enhanced in children due to increased nasal mucosal vascularization and faster absorption [25].

Pharmacodynamically, remimazolam exerts typical benzodiazepine anxiolytic and sedative effects by enhancing γ-aminobutyric acid (GABA)–mediated neurotransmission. The drug binds to the benzodiazepine α+/γ− site on the GABA-A receptor as a positive allosteric modulator, increasing channel opening frequency and chloride ion influx in the presence of endogenous GABA [26,27].

In adults, remimazolam has demonstrated a favorable safety profile, characterized by rapid recovery and a lower incidence of respiratory depression and hemodynamic instability. Nonetheless, post-marketing surveillance data, including analyses from the FEARS database, indicate that adverse effects may still occur, particularly in high-risk populations such as elderly patients and those with significant comorbidities [11]. In pediatric patients, the PD profile appears even more advantageous. A randomized study published in 2025 investigating the prevention of emergence delirium following laparoscopic surgery demonstrated that remimazolam significantly reduced PAED scores and the incidence of emergence delirium, while also improving hemodynamic stability (lower MAP and heart rate, higher SpO_2_), reducing postoperative pain, and shortening PACU length of stay [13].

From a clinical perspective, the PK/PD profile of remimazolam translates into rapid titratability, predictable recovery, and limited drug accumulation, features that are particularly advantageous in pediatric patients, in whom interindividual variability and vulnerability to prolonged sedation are major concerns (Table 1).

## 3. Clinical Applications

Principal clinical applications of remimazolam in pediatric anesthesia and sedation, encompass premedication, induction of general anesthesia, non-operating room procedures, and intensive care unit sedation (Table 2).

### 3.1. Premedication and Anesthesia Induction

In recent years, remimazolam has been introduced as an alternative to propofol for anesthetic induction, owing to its pharmacological profile characterized by rapid onset of action, predictable effects, and favorable hemodynamic stability [1,2].

Remimazolam is administered using a regimen that includes a loading dose followed by continuous infusion. This approach allows rapid attainment of predefined sedation targets according to the Richmond Agitation–Sedation Scale (RASS), typically ranging between −3 and 0 [28].

In the pediatric population, induction with remimazolam has been evaluated mainly through comparative studies with propofol. In a randomized trial published in 2025 involving 100 children undergoing adenotonsillectomy, remimazolam was shown to provide effective anesthetic induction, although with a slower onset compared with propofol (64.3 ± 8.1 vs. 38.3 ± 4.5 s) [29]. Despite this, extubation time and awakening time were significantly shorter in the remimazolam group (12.9 ± 2.2 vs. 14.5 ± 3.2 min; 19.9 ± 4.7 vs. 21.8 ± 4.5 min, respectively). Compared with propofol, remimazolam was also associated with a significantly lower incidence of hypotension and greater overall hemodynamic stability [28,30].

Premedication plays an important role in the quality of anesthetic induction in pediatric patients. A randomized trial published in 2024, including 90 children, compared intranasal remimazolam with dexmedetomidine as premedication before general surgery [15]. The authors reported that most children receiving intranasal remimazolam at a dose of 1.5 mg kg^−1^ experienced a significant reduction in preoperative anxiety within 10 min of administration. The estimated intranasal bioavailability of remimazolam is approximately 25–50%, indicating that clinically effective concentrations can be achieved rapidly even in the absence of intravenous access, making this route particularly advantageous in younger children.

Additional indirect evidence derives from a randomized controlled study investigating the prevention of emergence delirium after laparoscopic inguinal hernia repair [31]. Although not specifically designed to assess induction, the study demonstrated that remimazolam improved cardiocirculatory stability from the early phases of anesthesia. Patients receiving remimazolam showed lower mean arterial pressure and heart rate, higher oxygen saturation, reduced postoperative pain, shorter PACU stay, decreased sufentanil consumption, and greater parental satisfaction (all *p* < 0.05).

Across pediatric studies, no statistically significant differences have been observed in the incidence of respiratory depression when comparing remimazolam with propofol during induction [29]. In this study, remimazolam was used at an induction dose of 0.3 mg/kg over 60 s, and a maintenance dose of 1 mg/kg/h [29].

These findings suggest that remimazolam does not increase the risk of respiratory depression during the induction phase; however, they do not support the conclusion that such a risk is entirely absent.

Overall, available evidence indicates that remimazolam provides effective and hemodynamically stable induction of anesthesia in children, with a safety profile comparable to propofol. While onset of action may be slower, the balance between cardiovascular stability and recovery characteristics supports its consideration as a viable alternative in selected pediatric populations, since cardiorespiratory complications are among the most frequent in pediatric anesthesia [32].

### 3.2. Non-Operating Room Anesthesia

Non-operating room anesthesia (NORA) and procedural sedation in children outside the operating theater represent a significant clinical challenge. These settings require reliable sedation while ensuring spontaneous ventilation and hemodynamic stability, often in environments where airway management is more complex than in the operating room. In this context, the introduction of remimazolam has generated increasing interest because of its distinctive pharmacological profile [33,34].

In NORA settings, remimazolam has been primarily employed for diagnostic and minimally invasive procedures such as magnetic resonance imaging, computed tomography, gastrointestinal endoscopy, flexible bronchoscopy, and echocardiography. In all these scenarios, maintaining adequate sedation while preserving spontaneous respiration and cardiovascular stability is crucial. Evidence from prospective and observational studies suggests that remimazolam generally exhibits a more favorable safety profile than commonly used sedatives such as propofol and midazolam, being associated with a lower incidence of respiratory depression and cardiovascular compromise [35]. This hemodynamic and respiratory stability is particularly relevant in NORA procedures, where rapid access to the airway may be limited.

An additional advantage of remimazolam is the availability of flumazenil as a specific antagonist. The possibility of pharmacological reversal enhances safety, especially in outpatient and diagnostic settings. However, the literature emphasizes the need for careful post-reversal monitoring, as prolonged infusions may be associated with re-sedation or delayed respiratory depression [36,37].

Despite encouraging results, remimazolam is not yet approved for pediatric use in many regions, and currently available studies present methodological limitations. Nevertheless, clinical evidence indicates that remimazolam can be safely combined with other agents, such as low-dose propofol or opioid analgesics, to optimize sedation quality while reducing the incidence of adverse events and maintaining an acceptable safety profile in pediatric patients [38].

In this study, remimazolam was used at an induction dose of 12 mg/kg/h until a Ramsay sedation score of 3 was achieved, and maintenance was achieved with a dosage of 1–2 mg/kg/h [38].

Importantly, a study published in 2024 demonstrated that the exposure–response relationship of remimazolam differs between children and adults [39]. Specifically, children older than six years may require higher weight-adjusted doses to achieve adequate depth of sedation. These findings underscore the need for pediatric-specific dosing strategies rather than extrapolation from adult regimens.

Despite methodological heterogeneity, studies conducted in non-operating room settings consistently suggest that remimazolam allows effective procedural sedation while preserving spontaneous ventilation and cardiovascular stability, a combination that is particularly desirable in environments where airway management may be challenging. Overall, remimazolam represents a promising sedative option for pediatric NORA due to its titratability, favorable cardiorespiratory safety profile, and reversibility. Remimazolam may represent a valid option in NORA procedures where there is difficulty in airway management (airway endoscopies, digestive endoscopies and dental procedures). Another possible use concerns outpatient procedures, where the main causes of unplanned admission are pain and PONV [40,41].

However, caution is warranted given the heterogeneity of existing protocols, limited regulatory approval, and incomplete pediatric evidence.

### 3.3. Intensive Care Unit

Sedation management in the intensive care unit (ICU) represents one of the most complex and delicate aspects of care for critically ill patients. The goals of sedation extend beyond the mere control of agitation and include optimization of mechanical ventilation, reduction of oxygen consumption, prevention of neuroendocrine stress responses, and improvement of patient comfort. In recent years, ICU sedation strategies have progressively shifted toward lighter and more titratable approaches, with the aim of reducing the duration of mechanical ventilation and shortening ICU length of stay.

Traditionally used sedatives in this setting, such as propofol and midazolam, present well-recognized limitations. Propofol is associated with dose-dependent hypotension and respiratory depression [42] and, during prolonged infusions, with the risk of propofol infusion syndrome, a rare but potentially fatal condition characterized by multiorgan failure [43]. Midazolam, although generally associated with fewer cardiorespiratory depressive effects, is characterized by prolonged recovery times and longer durations of mechanical ventilation following continuous infusion [44,45]. In this context, remimazolam has emerged as a novel sedative developed to combine the PD advantages of benzodiazepines with a more favorable pharmacokinetic profile.

Remimazolam exerts its sedative effects through interaction with gamma-aminobutyric acid type A (GABAA) receptors [20,46], increasing neuronal chloride ion influx, inducing membrane hyperpolarization, and reducing neuronal excitability. Clinically, this results in sedation, anxiolysis, and anterograde amnesia [47,48].

Owing to the methyl-propionate side chain, remimazolam is rapidly metabolized by hepatic carboxylesterase 1 (CES1) into a pharmacologically inactive carboxylic acid metabolite, CNS7054. This metabolic pathway confers a particularly short elimination half-life, ranging between 5 and 10 min [39,49]. Following hydrolysis, metabolites undergo conjugation reactions and are subsequently eliminated via renal excretion. Importantly, remimazolam exhibits organ-independent metabolism, reducing the risk of drug accumulation in patients with hepatic or renal impairment [50]. This characteristic is of particular relevance in ICU patients, in whom multiorgan dysfunction is frequently observed. Pharmacokinetic studies further indicate that age and body weight do not significantly influence remimazolam metabolism [16]. In pediatric patients aged 2–6 years, pharmacokinetic parameters are comparable to those observed in adults [39].

The context-sensitive half-time (CSHT), defined as the time required for plasma drug concentration to decrease by 50% after discontinuation of an infusion, is a critical parameter for ICU sedation. Gao et al. analyzed the PK of remimazolam and CNS7054 in arterial blood samples from 24 children aged 3–6 years and reported a 4 h CSHT of 17 min [21]. This value is comparable to that of propofol, indicating a similarly rapid decline in drug concentration after infusion cessation. Based on these PK properties, remimazolam appears suitable for prolonged ICU sedation [51], without an increase in ICU mortality or adverse events when compared with propofol or midazolam [28,52].

In the ICU, invasive mechanical ventilation remains one of the main indications for pharmacological sedation, despite being associated with significant adverse effects [53,54]. Sedation must ensure patient comfort and ventilator tolerance while preventing patient–ventilator dyssynchrony, without inducing excessive sedation that could prolong weaning and increase complication rates. Contemporary ICU sedation strategies therefore emphasize light-to-moderate, titratable, and reversible sedation.

In studies evaluating remimazolam in ICU settings, sedation depth is routinely monitored using the Richmond Agitation–Sedation Scale (RASS), which allows real-time drug titration in mechanically ventilated patients. In remimazolam-based protocols, a RASS target between −3 and 0 corresponds to light-to-moderate sedation [51]. RASS scores are typically assessed every two hours; infusion rates are reduced if excessive sedation occurs (RASS −4), whereas inadequate sedation is managed with rescue boluses or infusions of propofol. The pharmacological profile of remimazolam—characterized by rapid onset, organ-independent metabolism, short duration of action, and a favorable safety profile—confers several advantages over conventional sedatives [55]. Moreover, Tang et al. [28] investigated remimazolam sedation in mechanically ventilated ICU patients following non-cardiac surgery and demonstrated that infusion rates of 0.125–0.15 mg·kg^−1^·h^−1^ achieved mild-to-moderate sedation. After the loading dose, mean arterial pressure decreased modestly from 97.3 ± 15.0 mmHg to 90.0 ± 17.3 mmHg, while heart rate remained stable (88.7 ± 20.1 bpm vs. 87.3 ± 19.6 bpm). Hypotension and tachycardia were the most frequent adverse events, whereas no deaths, serious adverse events, or treatment discontinuations were reported. Similarly, a prospective observational study by Yao et al. [52] involving 106 mechanically ventilated patients sedated for more than 24 h found no significant differences in ICU mortality or RASS scores between remimazolam, propofol, and midazolam. Notably, patients treated with remimazolam experienced a shorter duration of mechanical ventilation. In addition, the remimazolam group exhibited smaller alterations in acid–base balance and lactate levels, with no significant differences in renal, hepatic, or cardiac biomarkers.

Nevertheless, most PK studies of remimazolam in critically ill patients involve infusion durations not exceeding 24 h, leaving uncertainty regarding longer-term administration [56]. The Japanese ONO-2745-04 trial, designed to evaluate remimazolam for sedation during mechanical ventilation, was prematurely terminated due to unexpectedly high plasma concentrations observed in some patients after 24 h of infusion. However, subsequent work by Suzuki et al. [56] demonstrated that fixed-rate infusion (0.1 mg·kg^−1^·h^−1^), despite differing infusion durations between surgical and medical ICU populations, was not associated with time-dependent accumulation. Collectively, ICU studies suggest that remimazolam enables light-to-moderate, titratable sedation with limited hemodynamic impact and rapid offset, aligning well with contemporary sedation strategies aimed at minimizing ventilation duration and ICU length of stay. However, uncertainty remains regarding its optimal use during prolonged infusions and in specific pediatric subpopulations. In pediatric populations, evidence remains limited. A pediatric scoping review highlighted that although early signals of efficacy and safety exist, available studies are few, heterogeneous, and lack standardized dosing protocols [57]. A large cohort study involving 418 children reported successful induction (12 mg·kg^−1^·h^−1^) and maintenance of general anesthesia (1–2 mg·kg^−1^·h^−1^ with a 0.2 mg·kg^−1^ bolus) using remimazolam; however, 5% of patients required ephedrine for hypotension or bradycardia [58]. Two American case reports [59,60] described effective sedation and preserved spontaneous ventilation with continuous infusion rates of 15–20 μg·kg^−1^·min^−1^. Conversely, another case report documented apnea, bradypnea, oxygen desaturation, and hypotension in an obese child receiving remimazolam, all of which were rapidly reversible [61].

### 3.4. Analgesic Effect

**Figure 1:**
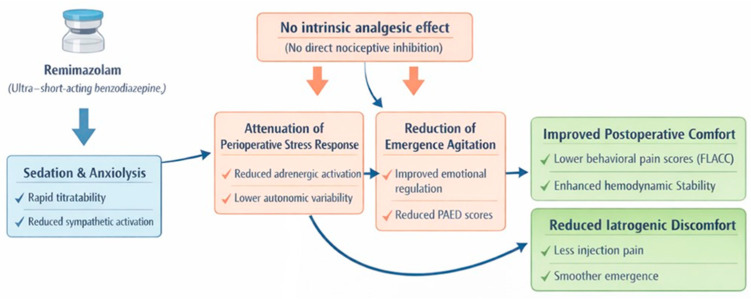
Indirect Analgesic and comfort-related effects of remimazolam in pediatric patients.

Although remimazolam lacks intrinsic analgesic properties, its indirect effects on postoperative comfort deserve consideration. Remimazolam, although it is a benzodiazepine devoid of intrinsic analgesic activity, shows in pediatric patients an indirect impact on postoperative pain through several clinically relevant mechanisms. A 2025 study highlighted that the administration of remimazolam during surgery was associated with significantly lower FLACC scores (a tool used for the assessment of behavioral pain) in the 10/20 min following extubation, suggesting better control of postoperative discomfort compared with control groups [13]. Despite the absence of direct analgesic action, clinical data suggests improved pain control in the immediate postoperative phase; this finding depends on the fact that the use of remimazolam is associated with a reduction in emergence agitation and with modulation of the perioperative stress response. The drug therefore appears to contribute indirectly to limiting emergence agitation by modulating the severity of postoperative pain [13]. In the same study, it emerges that the use of remimazolam is associated with a significant reduction in the pain component assessed through FLACC and with greater hemodynamic stability during the postoperative awakening phase, observing lower variability in heart rate and blood pressure thanks to inhibition of sympathetic nervous system activity [62]. This effect demonstrates the indirect influence that the drug exerts on pain perception through attenuation of the adrenergic response to the surgical stimulus. A further comparative study on anesthesiologic protocols in the pediatric setting demonstrated that remimazolam, compared with propofol, is associated with a marked reduction in injection pain [29] (4% vs. 48%). Obviously, in this case this refers to a circumscribed and procedural form of pain; however, it demonstrates that the drug is associated with reduced activation of iatrogenic nociceptive stimuli, an aspect particularly important in pediatric patients. In a review of clinical protocols for pediatric ENT procedures, an observational study emerges in which the use of remimazolam during adenotonsillectomy was evaluated. This study highlights that remimazolam, compared with propofol, is associated with a lower incidence of emergence agitation and with better postoperative pain control, with a clinically relevant reduction in painful symptomatology even in a context normally characterized by high pain intensity [35]. Remimazolam is not associated with a direct analgesic effect but rather with a better postoperative comfort profile, in turn associated with greater hemodynamic stability and reduced emergence agitation, a factor known to amplify pain perception in the pediatric population (Figure 1).

Although remimazolam lacks intrinsic analgesic properties, its sedative and anxiolytic effects contribute to attenuation of the perioperative stress response and reduction in emergence agitation. These mechanisms indirectly improve postoperative comfort, reduce behavioral pain expression, and enhance hemodynamic stability during recovery, thereby modulating pain perception in pediatric patients (Edited with GPT 5.2).

## 4. Adverse Effects

Despite exhibiting an overall favorable pharmacological profile, remimazolam is not devoid of critical issues, particularly regarding the occurrence of adverse events when administered to fragile populations such as pediatric patients. It is important to note that remimazolam is not indicated for pediatric patients. However, most of the medications we need to use to provide adequate patient care are not indicated for children, according to the product information.

The most frequently reported adverse events include hypotension, bradycardia, injection site pain, respiratory depression, postoperative nausea and vomiting (PONV), and emergence agitation. These effects were systematically described in the 2025 randomized clinical trial by Wu et al. [29], which investigated remimazolam in preschool-aged children undergoing adenotonsillectomy. Notably, despite the broad spectrum of reported adverse events, the incidence of emergence agitation, hypotension, and injection site pain was significantly lower in the remimazolam group compared with the propofol group (12% vs. 30%, *p* = 0.027; 26% vs. 48%, *p* = 0.023; 4% vs. 48%, *p* < 0.001). No statistically significant differences were observed between groups in the incidence of hemodynamic instability, nausea, or vomiting.

Consistent findings emerged from a 2025 retrospective observational study involving 193 children undergoing tonsillectomy or adenotonsillectomy [14]. In this cohort, patients receiving remimazolam experienced a significantly lower incidence of hypotension and bradycardia compared with those treated with propofol (2% vs. 7% and 1% vs. 5%, respectively). Overall, in the pediatric surgical setting, remimazolam appears to be associated with greater hemodynamic stability, faster postoperative recovery, and a reduced burden of perioperative complications.

Further evidence derives from a 2025 randomized trial including 184 pediatric patients aged 3–14 years undergoing laparoscopic inguinal hernia repair [13]. In this study, remimazolam was associated with improved hemodynamic stability, defined as mean arterial pressure and heart rate fluctuations below 10%, higher oxygen saturation values (>94%), reduced postoperative pain, shorter PACU stay, decreased sufentanil consumption, and greater parental satisfaction (all *p* < 0.05).

Additional safety data were reported by Kimoto et al. [58] in a large cohort study including 418 pediatric patients. Although mean arterial pressure variations exceeding 20% from baseline were observed in 75.2% of patients, only 5% required pharmacological intervention with ephedrine, suggesting that most hemodynamic fluctuations were transient and clinically well tolerated. The reported incidence of PONV was 13%, markedly lower than rates generally described in pediatric anesthesia [63]. However, the authors emphasized that the precise attribution of adverse events was limited by potential confounding factors inherent to the observational design.

Adverse effects related to alternative routes of administration have also been described. In a randomized clinical trial conducted in 2024 [15] comparing intranasal remimazolam and dexmedetomidine in 90 pediatric patients, remimazolam was associated with reduced cooperation during intranasal administration, likely due to nasal mucosal irritation, with statistically significant differences compared with control groups (*p* = 0.001 vs. group C; *p* = 0.010 vs. group D). Although remimazolam achieved a rapid reduction in preoperative anxiety—within 10 min of administration—the resulting sedation was mild and accompanied by notable local discomfort. Systemic adverse events were minimal: two episodes of bradycardia occurred in the dexmedetomidine group (one requiring atropine), whereas only one case of mild nausea and vomiting was reported in the remimazolam group.

The cardiovascular safety profile of remimazolam has been primarily characterized in adult populations, with relevant implications for pediatric use. In healthy volunteers, continuous intravenous infusion of remimazolam resulted in a reduction in mean arterial pressure of up to 20 mmHg, while systolic arterial pressure remained consistently above 80 mmHg [22]. Compared with total intravenous anesthesia using propofol, the combination of remimazolam and fentanyl has been associated with a lower incidence of postoperative hypotension, defined as a mean arterial pressure below 65 mmHg for at least one minute [64]. In elderly patients, comparative trials demonstrated a lower risk of postoperative hypotension (risk ratio 0.41, 95% CI 0.27–0.62) and bradycardia (risk ratio 0.58, 95% CI 0.34–0.98), without clinically relevant alterations in arterial pressure [65].

A meta-analysis focusing on endoscopic procedures confirmed that remimazolam is associated with a significantly reduced risk of hypotension (risk ratio 0.43, 95% CI 0.35–0.51) and bradycardia (risk ratio 0.42, 95% CI 0.38–0.58) compared with propofol [66]. No clinically meaningful effects on cardiac conduction, including PR interval or QRS duration, were observed [67].

In pediatric populations, a meta-analysis by Wang et al. including 309 patients (https://doi.org/10.1186/s40001-025-02383-z) found no significant difference in the risk of bradycardia between remimazolam and control groups (RR 0.85; 95% CI 0.34–2.12; *p* = 0.72), nor in the incidence of PONV (RR 1.06; 95% CI 0.74–1.51; *p* = 0.77) [68].

Hypersensitivity reactions to remimazolam appear to be rare but clinically relevant. A large review documented only ten cases of anaphylaxis among 6808 exposed patients [67]. Presentations were predominantly characterized by severe hypotension, with cardiopulmonary resuscitation required in two cases; cutaneous manifestations such as flushing or facial erythema were reported in four patients. Analysis of the FAERS database by Ye et al. identified significant safety signals for immune-mediated reactions, including anaphylactic shock and laryngeal edema, with immune system disorders showing a reporting odds ratio of 10.02 [69]. These findings underscore the importance of careful allergy assessment and immediate availability of adrenaline during clinical use.

Although remimazolam is often considered to confer a lower risk of respiratory depression compared with other sedatives, pharmacovigilance data indicate that respiratory complications remain clinically relevant. Significant safety signals involving the respiratory, cardiovascular, and immune systems have been reported [55]. Hypoventilation emerged as the adverse event with the strongest disproportionality signal, and reductions in oxygen saturation were reported at a markedly higher frequency than expected [69]. These observations support the need for vigilant monitoring and structured risk mitigation strategies, particularly in vulnerable populations.

Conversely, in controlled pediatric trials, severe respiratory events appear to be uncommon. In the study by Wu et al., respiratory depression occurred in 1–2% of patients in both treatment groups, without statistically significant differences [29]. Importantly, no episodes of excessive sedation were recorded 30 min after awakening in the remimazolam group. Similar findings were reported by Wan et al., with no cases of laryngospasm or bronchospasm and no clinically relevant differences in breath-holding or oxygen desaturation [14]. Overall, the incidence of respiratory events was low. Finally, in a study on preoperative intravenous sedation by Chen et al. [70], only a single episode of hypoxemia was observed, which resolved promptly with a simple mandibular support maneuver and did not require additional intervention. In summary, pediatric data suggests a generally favorable safety profile for remimazolam when used within controlled anesthetic and sedative settings. Nevertheless, real-world pharmacovigilance signals highlight the importance of careful patient selection, vigilant monitoring, and structured risk mitigation strategies, particularly in vulnerable populations (Table 3).

## 5. Conclusions

Remimazolam emerges as a promising sedative and anesthetic agent in pediatric practice, owing to its ultra-short-acting profile, organ-independent metabolism, predictable recovery, and availability of a specific antagonist. Across different clinical settings, including anesthetic induction, NORA, and ICU sedation, current evidence suggests that remimazolam provides effective sedation with greater hemodynamic stability and rapid offset compared with traditional agents such as propofol and midazolam. Although it lacks intrinsic analgesic properties, remimazolam appears to indirectly improve postoperative comfort by reducing emergence agitation and stress-related responses. Moreover, pediatric studies indicate a generally favorable safety profile when the drug is used in controlled settings; however, its use remains off-label in many countries, and available data are limited by heterogeneous protocols and small sample sizes. Further well-designed randomized trials are required to define optimal pediatric dosing strategies, long-term safety, and its precise role in pediatric anesthesia and sedation. In particular, studies on the most vulnerable pediatric populations (such as patients with cardiac diseases) would be desirable, and they could benefit from a safe and easy-to-use drug.

## Figures and Tables

**Table 1:** Pharmacological profile of remimazolam and comparison with conventional sedatives.

Feature	Remimazolam	Propofol	Midazolam
Drug class	Benzodiazepine (soft drug)	Hypnotic agent	Benzodiazepine
Onset of action	Rapid	Very rapid	Moderate
Offset/recovery	Rapid and predictable	Rapid	Prolonged
Metabolism	Ester hydrolysis (CES1)	Hepatic	Hepatic
Organ-independent metabolism	Yes	No	No
Accumulation risk	Minimal	Moderate (long infusions)	High
Hemodynamic stability	High	Moderate–low	Moderate
Respiratory depression	Low–moderate	Moderate–high	Moderate
Specific antagonist	Yes (flumazenil)	No	Yes (flumazenil)

**Table 2:** Clinical applications of remimazolam in pediatric patients.

Setting	Main Indication	Key Findings	Clinical Implications
Anesthetic induction	General anesthesia	Effective induction, slower onset than propofol, faster recovery	Alternative in hemodynamically fragile children
Premedication	Anxiety reduction	Rapid anxiolysis via intranasal route	Useful when IV access is difficult
NORA	Diagnostic/procedural sedation	Preserved spontaneous ventilation, stable hemodynamics	Advantageous in limited airway-access settings
ICU sedation	Mechanical ventilation	Light-to-moderate sedation, rapid offset	Compatible with modern light-sedation strategies

Abbreviations: IV, intravenous; NORA, non-operating room anesthesia; ICU, intensive care unit.

**Table 3:** Safety profile of remimazolam in pediatric studies.

Adverse Event	Frequency	Comparison with Propofol	Notes
Hypotension	Low–moderate	Lower	Usually transient
Bradycardia	Low	Similar or lower	Rare need for intervention
Respiratory depression	Low	Similar	Requires monitoring
Emergence agitation	Reduced	Lower	Clinically relevant benefit
PONV	Low	Similar or lower	Favorable profile
Hypersensitivity	Rare	Rare	Vigilance required

## Data Availability

No new data were created or analyzed in this study.
